# Microglia and motor neurons during disease progression in the SOD1^G93A^ mouse model of amyotrophic lateral sclerosis: changes in arginase1 and inducible nitric oxide synthase

**DOI:** 10.1186/1742-2094-11-55

**Published:** 2014-03-23

**Authors:** Katherine E Lewis, Anna L Rasmussen, William Bennett, Anna King, Adrian K West, Roger S Chung, Meng Inn Chuah

**Affiliations:** 1 Wicking Dementia Research and Education Centre, University of Tasmania, Hobart, TAS, Australia; 2 School of Medicine, University of Tasmania, Hobart, TAS, Australia; 3 Australian School of Advanced Medicine, Macquarie University, Sydney, NSW, Australia

**Keywords:** Amyotrophic lateral sclerosis, Microglia, Inducible nitric oxide synthase, Arginase1, Motor neurons, Lumbar spinal cord, Cervical spinal cord, Neuroinflammation

## Abstract

**Background:**

Amyotrophic lateral sclerosis (ALS) is a fatal neurodegenerative disease affecting the motor system. Although the etiology of the disease is not fully understood, microglial activation and neuroinflammation are thought to play a role in disease progression.

**Methods:**

We examined the immunohistochemical expression of two markers of microglial phenotype, the arginine-metabolizing enzymes inducible nitric oxide synthase (iNOS) and arginase1 (Arg1), in the spinal cord of a mouse model carrying an ALS-linked mutant human superoxide dismutase transgene (SOD1^G93A^) and in non-transgenic wild-type (WT) mice. Immunolabeling for iNOS and Arg1 was evaluated throughout disease progression (6 to 25 weeks), and correlated with body weight, stride pattern, wire hang duration and ubiquitin pathology. For microglia and motor neuron counts at each time point, SOD1^G93A^ and WT animals were compared using an independent samples *t*-test. A Welch *t*-test correction was applied if Levene’s test showed that the variance in WT and SOD1^G93A^ measurements was substantially different.

**Results:**

Disease onset, measured as the earliest change in functional parameters compared to non-transgenic WT mice, occurred at 14 weeks of age in SOD1^G93A^ mice. The ventral horn of the SOD1^G93A^ spinal cord contained more microglia than WT from 14 weeks onwards. In SOD1^G93A^ mice, Arg1-positive and iNOS-positive microglia increased 18-fold and 7-fold, respectively, between 10 and 25 weeks of age (endpoint) in the lumbar spinal cord, while no increase was observed in WT mice. An increasing trend of Arg1- and iNOS-expressing microglia was observed in the cervical spinal cords of SOD1^G93A^ mice. Additionally, Arg1-negative motor neurons appeared to selectively decline in the spinal cord of SOD1^G93A^ mice, suggesting that Arg1 may have a neuroprotective function.

**Conclusions:**

This study suggests that the increase in spinal cord microglia occurs around and after disease onset and is preceded by cellular pathology. The results show that Arg1 and iNOS, thought to have opposing inflammatory properties, are upregulated in microglia during disease progression and that Arg1 in motor neurons may confer protection from disease processes. Further understanding of the neuroinflammatory response, and the Arg1/iNOS balance in motor neurons, may provide suitable therapeutic targets for ALS.

## Introduction

Amyotrophic lateral sclerosis (ALS) is a progressive neurodegenerative disease affecting the motor system. In ALS, spinal cord motor neurons degenerate and die, causing progressive paresis and paralysis leading to death due to respiratory failure within 2 to 5 years of diagnosis. Disease etiology is not fully understood, and there is only one therapeutic (Riluzole) shown to extend ALS patient lifespan for a matter of months [1]. It is therefore important to characterize the processes involved in disease progression in order to identify potential new therapeutic targets.

A key discovery was the identification of mutations in the antioxidant enzyme Cu/Zn superoxide dismutase 1 (SOD1) gene as the cause of approximately 20% of familial ALS [2]. SOD1 mutations lead to a toxic gain of function, triggering a cascade of events that lead to the degeneration of motor neurons [3]. Expression of a mutant human SOD1 transgene in mice (SOD1^G93A^ mice) results in similar pathology and symptoms to human ALS [4]; SOD1^G93A^ mice are currently the most widely used ALS mouse model.

Although ALS is characterized primarily by selective degeneration of motor neurons, it is increasingly regarded as a complex multifactorial disease involving dysregulation of cellular processes affecting both neurons and glial cells [5-7]. Two proposed pathogenic mechanisms in ALS are oxidative stress and neuroinflammation. Biochemical markers of oxidative injury are increased in tissue specimens from ALS patients, while SOD1^G93A^ mice show elevated levels of protein oxidation and nitrosylation, and lipid oxidation [8,9]. Microglial activation is apparent in tissue from ALS patients [10], and is also readily apparent in SOD1^G93A^ mice [11], with a switch from the neuroprotective M2-like microglial phenotype to the neurotoxic M1-like microglial phenotype associated with the transition of SOD1^G93A^ mice from a stable to a rapidly progressive phase of disease [12,13].

The metabolism of nitric oxide (NO) may provide a link between microglial activation and oxidative injury to motor neurons: microglial-mediated damage to motor neurons in cell culture is partially dependent on the production of microglial NO [14]. The mutant SOD1 may have the capacity to catalyze the production of reactive oxygen species such as peroxynitrite [15], also contributed to by increased cellular expression of inducible nitric oxide synthase (iNOS) [16]. In microglia, the production of NO is regulated by the relative amounts of two arginine-metabolizing enzymes, arginase 1 (Arg1) and inducible nitric oxide synthase (iNOS) - Arg1 converts arginine into ornithine and urea, while iNOS converts arginine into citrulline and produces NO [17]. iNOS protein expression in SOD1^G93A^ mice has been reported in glial cells especially in the ventral horn [18], while immunoreactivity for Arg1 has been demonstrated in motor neurons in SOD1^G93A^ and non-transgenic mice [19]. As microglial expression of Arg1 and iNOS varies with M1 and M2 microglial phenotypes [20], the production of NO may play an important role in the putative M2-to-M1 switch and subsequent toxicity to motor neurons [13]. Whilst iNOS and Arg1 act on the same substrate but lead to opposing outcomes [17], they have not been investigated in microglia and motor neurons in the same experimental preparation.

In this study we characterized changes in lumbar and cervical spinal cord microglia in terms of Arg1 and iNOS protein expression, from pre-symptomatic to end-stage in the SOD1^G93A^ mice. We have temporally compared microglial changes with the onset of functional deficits, and with the appearance of spinal cord ubiquitin pathology in these mice. Additionally, we have examined expression of Arg1 in lumbar motor neurons. Our findings demonstrate increasing expression of Arg1 and iNOS protein, reflecting the complex role of inflammation in ALS disease progression.

## Materials and methods

### Ethics statement

All procedures involving animals were approved by the University of Tasmania Animal Ethics Committee (permit numbers A10995 and A11958) and were consistent with the Australian Code of Practice for the Care and Use of Animals for Scientific Purposes. SOD1^G93A^ mice with advanced symptoms of ALS had food placed on bedding, to ensure easy access and continued nutrition. All perfusions were carried out under terminal sodium pentobarbitone anesthesia when all reflexes were absent.

### Animals

SOD1^G93A^ transgenic mice (B6.Cg-Tg(SOD1*G93A)1Gur/J, Jackson Laboratories, Bar Harbor, ME, USA) and wild-type littermates (WT; C57BL/6) were maintained in a 12 hour light: dark cycle in filtered, sterile air by the University of Tasmania Animal Services. All mice were housed in OptiMICE cages with standard bedding, and were provided with food and water *ad libitum*. Presence of the SOD1^G93A^ transgene was assessed according to standard protocols [21].

### Reagents

Nitric acid, sodium azide, paraformaldehyde, urea, citric acid and sodium citrate were obtained from Fluka and Sigma (Sigma-Aldrich, St Louis, MO, USA). Biotinylated tomato lectin (TL), normal goat serum, the Mouse on Mouse (MOM) reagent kit, and the streptavidin/biotin blocking kit were obtained from Vector Laboratories (Burlingame, CA, USA). The following primary antibodies were used: anti-ionized calcium binding adaptor molecule 1 (Iba1, Wako, Osaka, Japan); anti-iNOS (Santa Cruz Biotechnology, Dallas, TX, USA); anti-Arg1 (Santa Cruz Biotechnology); anti-ubiquitin (Dako, Carpinteria, CA, USA); and anti-dephosphorylated neurofilament heavy and medium chain (SMI32, Covance, Princeton, NJ, USA) (Table 1). AlexaFluor-conjugated and biotin-conjugated secondary antibodies, AlexaFluor-conjugated streptavidin, and Nuclear Yellow dye were obtained from Molecular Probes (Life Technologies, Carlsbad, CA, USA) (Table 1).

**Table 1:** Antibodies and detection reagents for immunohistochemistry

**Antibody/detection reagent**	**Type**	**Dilution**	**Incubation**
Anti-Iba1	Rabbit polyclonal	1:500	2 hours, RT
Anti-iNOS	Mouse monoclonal	1:100	2 hours, RT
Anti-arginase1	Mouse monoclonal	1:100	O/N, 4°C
Anti-ubiquitin	Rabbit polyclonal	1:1000	O/N, RT
Anti-dephosphorylated neurofilament (SMI32)	Mouse monoclonal	1:2000	O/N, RT
*Anti-rabbit IgG, biotinylated	Goat polyclonal	1:500	1 hour, RT
*Anti-rabbit IgG, AlexaFluor^®^594-conjugated	Goat polyclonal	1:1000	2 hours, RT
*Anti-mouse IgG, AlexaFluor^®^488-conjugated	Goat polyclonal	1:1000	2 hours, RT
*MOM kit anti-mouse IgG, biotinylated	MOM kit	1:250	20 minutes, RT
Streptavidin, AlexaFluor^®^488-conjugated	Streptavidin	1:500	1 hour, RT
Streptavidin, AlexaFluor^®^594-conjugated	Streptavidin	1:500	2 hours, RT
Nuclear yellow	Nuclear dye	1:10000	20 minutes, RT

*Secondary antibodies. Iba1, ionized calcium binding adaptor molecule 1; iNOS, inducible nitric oxide synthase; O/N, overnight; MOM, Mouse on Mouse; RT, room temperature.

### Preparation of time-series spinal cord tissue from SOD1^G93A^ and wild-type mice

#### ***Time points***

Spinal cord samples were obtained from SOD1^G93A^ mice and their WT littermates at 6, 10, 14, 18 and 22 weeks of age, and at disease endpoint. Disease endpoint, measured as a 20% reduction from pre-disease maximum body weight, typically occurred at 25 weeks of age in SOD1^G93A^ mice, with WT mice at 25 weeks of age used for comparison. For each of the above time points, lumbar spinal cord samples were obtained from three animals per genotype per time point (a mixture of male and female mice), with the exception of two WT mice at 6 weeks of age, and at 22 weeks of age when four animals per genotype were used. For an additional comparison, cervical spinal cord was obtained from two animals per genotype at 10 weeks of age, three WT and two SOD1^G93A^ mice at 14 weeks of age, and three animals per genotype at 18 and 22 weeks of age.

#### ***Perfusion and tissue preparation***

At the appropriate time point, animals were deeply anesthetized with sodium pentobarbitone (60 mg/kg, intraperitoneally). When all reflexes were absent, mice were transcardially perfused with 10 mL 10 mM PBS followed by 20 mL 4% w/v paraformaldehyde (PFA) in PBS. The T12-L1 vertebrae, containing the L2-L5 lumbar spinal cord segment, were dissected and post-fixed in 4% PFA overnight at 4°C, then stored in PBS with 0.01% w/v sodium azide. Cervical vertebrae, from the subset of animals described above, were collected in the same manner. All samples were incubated in rapid decalcification solution (5% v/v nitric acid with 0.05% w/v urea) for 2 hours at room temperature, then washed thoroughly with distilled water. Decalcified samples were dehydrated and embedded in paraffin wax using an automated tissue processor (Leica Biosystems ASP200-S, Melbourne, Australia). Sections (4 μm) were cut on a microtome (Microm HM325, Walldorf, Germany), mounted on Flex slides (Dako), dried at 37°C overnight and then stored at room temperature. Prior to immunohistochemistry, paraffin-embedded sections were heated to 60°C for 10 minutes for efficient paraffin removal, dewaxed in xylene and rehydrated through a series of graded ethanols to distilled water.

### Immunolabeling for microglial markers, inducible nitric oxide synthase and arginase1 in paraffin-embedded SOD1^G93A^ and wild-type spinal cord tissue

Spinal cord microglia were identified either by anti-Iba1 immunolabeling or by TL labeling [22,23]. TL labeling was also observed in some endothelial cells and motor neurons, which could be distinguished from microglia by their different size and morphology. iNOS and Arg1 are considered putative M1 and M2 microglial markers, respectively [20]. Immunostaining for Iba1 and for Arg1 was preceded by antigen retrieval. Antigen retrieval was performed by heating slides in 10 mM citrate buffer, pH 6 (2 mM citric acid, 8 mM sodium citrate) in a pressure cooker (Russell Hobbs, Braeside, Australia) at 100% power for 6 minutes followed by 60% power for 14 minutes, then cooled to room temperature and rinsed in PBS.

All immunostaining steps were carried out in a humidified light-safe chamber at room temperature unless otherwise specified; slides were washed three times in PBS between steps. To detect Iba1, sections were blocked with 10% v/v normal goat serum in PBS for 1 hour, then incubated with anti-Iba1, biotinylated goat anti-rabbit secondary antibody, streptavidin-AlexaFluor488 and Nuclear Yellow in PBS (Table 1). To detect Arg1 and iNOS, the MOM kit was used according to the manufacturer’s instructions. Briefly, sections were blocked with MOM blocking reagent for 1 hour and rinsed with PBS and MOM diluent; primary antibody (Arg1 or iNOS) was applied in MOM diluent, then detected with MOM biotinylated secondary antibody and streptavidin-AlexaFluor488 (Table 1). Following Arg1 or iNOS labeling, any free binding sites were blocked using the streptavidin/biotin blocking kit according to the manufacturer’s instructions; sections were then incubated with biotinylated TL (1:500, 1 hour), streptavidin-AlexaFluor594, and Nuclear Yellow (Table 1). All slides were coverslipped with aqueous fluorescence mounting medium (Dako).

### Microglial image analysis and cell counts

Images were captured with an Olympus BX50 fluorescence microscope fitted with a CoolSnapHQ2 camera (Photometrics, Tucson, AZ, USA). Immunohistochemistry counts for Iba1-, TL-, Arg1- and iNOS-positive cells were performed on three cross sections from the lumbar spinal cord, each separated by a minimum distance of 45 μm. The spinal cord ventral horn was defined as the region of gray matter on the ventral side of a horizontal line crossing through the central canal, bounded by a vertical midline through the sagittal plane. Images of the ventral horn were captured at 400 × magnification and cells were counted using ImageJ software (National Institutes of Health, Bethesda, MD, USA) and Adobe CS6 (Adobe Systems Incorporated, San Jose, CA, USA). Counts from the right and left ventral horns of each section were averaged and divided by the area in square millimeters. To avoid pseudoreplication, the average count from three sections was used as a single measurement per animal for statistical analysis. At each time point, SOD1^G93A^ and WT animals were compared using an independent samples *t*-test (SPSS Statistics 20, IBM Corp., Armonk, NY, USA). A Welch *t*-test correction was applied if Levene’s test showed that the variance in WT and SOD1^G93A^ measurements was substantially different. Data are presented as mean ± standard error of the mean, and the statistical test used for the comparison is indicated (*t*-test = independent samples *t*-test for equal variances; Welch *t*-test = independent samples *t*-test for unequal variances), with *P* < 0.05 considered significant.

Semi-quantitative analysis of the expression level of Arg1 and iNOS in SOD1^G93A^ microglia was performed in a subset of microglia. For both Arg1 and iNOS, one section was randomly selected from each animal at 6 weeks of age (n = 3) and at 22 weeks of age (n = 4). The cell bodies of eight microglia, showing the strongest Arg1 or iNOS staining in these sections, were outlined in ImageJ. The percentage of the cell body area showing positive immunostaining for Arg1 or iNOS was then calculated. To account for any variation in immunostaining intensity and background fluorescence between sections, positive immunostaining in 8-bit black and white images was defined as any pixels having a gray value greater than 3.5 standard deviations above the mean gray value of the whole image.

### Immunolabeling for ubiquitin in paraffin-embedded SOD1^G93A^ and wild-type spinal cord tissue

To determine the age at which neuronal pathology in the SOD1^G93A^ mice became evident, we immunostained for ubiquitin in paraffin-embedded lumbar spinal cords (6, 10, 14 and 25 weeks of age), and cervical spinal cords (10, 14, 18 and 22 weeks of age). Dephosphorylated neurofilament (SMI32) was used as a motor neuron marker to examine the localization of ubiquitin pathology. Anti-ubiquitin and SMI32 were applied overnight and detected with anti-rabbit-AlexaFluor594 and anti-mouse-AlexaFluor488 (Table 1). Immunostaining was examined using a fluorescence microscope (Leica DM LB2) and images were acquired on a Clara CCD cooled camera (Andor Technology, Belfast, UK) using NIS-Elements D software (Nikon, Tokyo, Japan).

### Assessment of functional and behavioral changes in SOD1^G93A^ and wild-type mice

#### ***Functional assessment of mice***

Seventeen SOD1^G93A^ and 13 WT female mice were assessed from pre-symptomatic stages through to 25 weeks (disease endpoint). Body weight was measured at least every 3 days, and daily when the mice were nearing disease endpoint. The wire-hang test, used to assess the ability to hang on to a wire cage lid when inverted (up to a maximum of 60 seconds), was measured once per week and the longest hang duration of three attempts was recorded. Stride pattern was examined once per week, with front (red) and rear (blue) nontoxic paint (GlobalColours, Marrickville, Australia) paw prints recorded as the mice walked along a 60 cm length of paper. Parameters measured from the stride pattern were stride length (distance between consecutive rear paw prints on the same side), hind-base width (lateral distance between hind paws), front-base width (lateral distance between front paws) and uniformity measurement (difference in placement of the hind paw compared with that of the front paw from the previous step) [24].

#### ***Statistical analysis of functional data***

Body weight and stride test measurements were averaged so each mouse had one measurement per week. WT and SOD1^G93A^ groups were compared using a Mann Whitney *U* test (MWU) for nonparametric data (SPSS Statistics 20). All data are presented as mean ± standard error of the mean, with *P* < 0.05 considered significant.

## Results

### SOD1^G93A^ mice show an increasing number of microglial cells in the lumbar ventral horn as disease progresses

**Figure 1:**
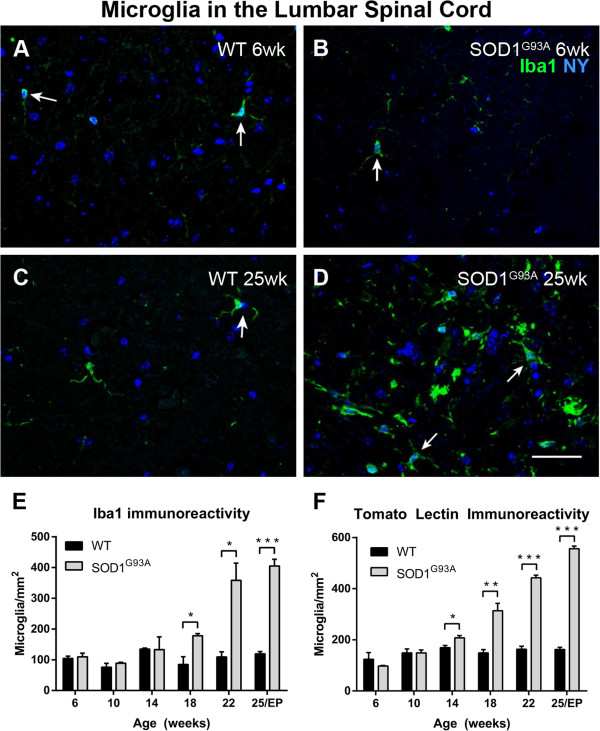
**Anti-ionized calcium binding adaptor molecule 1 (Iba1) and tomato lectin labeling in amyotrophic lateral sclerosis-linked mutant human superoxide dismutase transgene (SOD1**^**G93A**^**) and wild-type (WT) lumbar spinal cord ventral horn.** Few Iba1-positive microglia (arrows in A-D) were present in the ventral horn at 6 weeks of age in WT **(A)** and SOD1^G93A ^**(B)** mice. The number of microglia was not increased at 25 weeks of age in WT mice **(C)**, but was substantially increased at 25 weeks (disease endpoint, EP) in SOD1^G93A^ mice **(D)**. Microglial labeling with Iba1 **(E)** and tomato lectin **(F)** increased with disease progression. Scale bar 50 μm in A-D. *P < 0.05, **P < 0.01, ***P < 0.001. NY, Nuclear yellow.

Both Iba1 and TL labeling showed an increase in the number of microglial cells in the ventral horn of the lumbar spinal cord, at the symptomatic and end-stages of disease in SOD1^G93A^ mice (Figure 1; arrows in Figure 1A-D indicate microglia labeled with Iba1). Iba1 immunolabeling revealed an increased number of microglia in the lumbar ventral horn of SOD1^G93A^ mice compared to WT mice at 18 weeks of age (178 ± 7 microglia/mm^2^ versus 85 ± 26 microglia/mm^2^, *t*-test *P* = 0.025), with the number of microglia in SOD1^G93A^ mice continuing to increase until disease endpoint (Figure 1E). More intense labeling was observed in microglia with TL than with Iba1. Given this increased sensitivity, the number of TL-labeled microglia in SOD1^G93A^ mice (208 ± 9 microglia/mm^2^) was greater than that of WT mice (169 ± 8 microglia/mm^2^, *t*-test *P* = 0.034) beginning at 14 weeks of age and thereafter (Figure 1F). Thus, by both measures of microglial number, Iba1-immunolabeling and TL-labeling, there was a large increase in the number of microglia in SOD1^G93A^ spinal cords from 14 weeks of age onwards.

### SOD1^G93A^ mice show an increasing number of arginase1-positive microglia in the lumbar ventral horn as disease progresses

**Figure 2:**
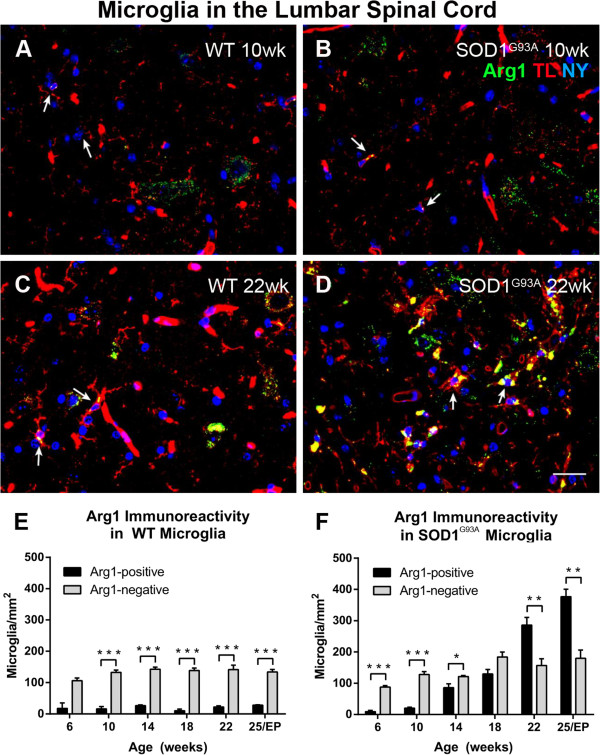
**Arginase1 (Arg1) expression in microglia of amyotrophic lateral sclerosis-linked mutant human superoxide dismutase transgene (SOD1**^**G93A**^**) and wild-type (WT) lumbar spinal cord ventral horn.** A subset of ventral horn tomato lectin (TL)-positive (red) microglia expressed Arg1 (green) (arrows, A-D) at 10 weeks of age in both WT **(A)** and SOD1^G93A^ **(B)** mice. The number of microglia expressing Arg1 was unchanged at 22 weeks of age in WT mice **(C)**; in contrast, 22-week SOD1^G93A^ spinal cord **(D)** showed a far greater number of Arg1-positive microglia. The numbers of Arg1-negative and Arg1-positive microglia remained constant over time in WT mice **(E)**. The number of Arg1-positive microglia increased with disease progression in SOD1^G93A^ mice **(F)**. Scale bar 50 μm in A-D. *P < 0.05, **P < 0.01, ***P < 0.001. NY, Nuclear yellow; EP, endpoint

Immunoreactivity for Arg1 was present in a subset of microglial cells within the lumbar ventral horn in both SOD1^G93A^ and WT mice (Figure 2A-D, arrows). In WT mice, the numbers of Arg1-positive and Arg1-negative microglia remained relatively constant over time (Figure 2E), with more Arg1-negative than Arg1-positive microglia at all time points (Welch *t*-test *P* = 0.079 at 6 weeks; *t*-test *P* < 0.05 at 10 to 25 weeks). In SOD1^G93A^ mice the number of Arg1-negative microglia increased slightly over time (Figure 2F). At 6 and 10 weeks of age, the SOD1^G93A^ lumbar ventral horn showed more Arg1-negative than Arg1-positive microglia, a pattern similar to that found in the WT ventral horn. However, the number of Arg1-positive microglia in SOD1^G93A^ mice increased progressively from 14 weeks of age (86 ± 12 microglia/mm^2^) to 25 weeks (377 ± 24 microglia/mm^2^). By 22 and 25 weeks, the number of Arg1-positive microglia was greater than the number of Arg1-negative microglia in the SOD1^G93A^ lumbar ventral horn (22 weeks: 286 ± 29 Arg1-positive microglia/mm^2^ versus 157 ± 22 Arg1-negative microglia/mm^2^, *t*-test *P* = 0.006; 25 weeks: 377 ± 24 Arg1-positive microglia/mm^2^ versus 180 ± 27 Arg1-negative microglia/mm^2^, *t*-test *P* = 0.005) (Figure 2F). At endpoint, there was an 18-fold increase in Arg1-positive microglia when compared to 10 weeks of age (Figure 2F).

### SOD1^G93A^ mice show an increasing number of inducible nitric oxide synthase-expressing microglia in the lumbar ventral horn as disease progresses

**Figure 3:**
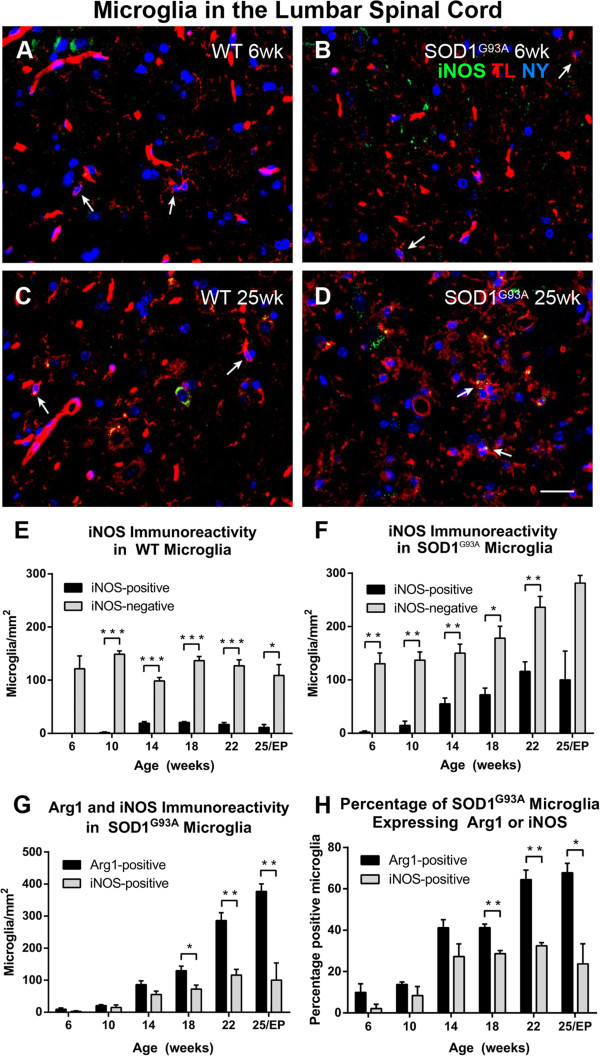
**Inducible nitric oxide synthase expression in microglia of amyotrophic lateral sclerosis-linked mutant human superoxide dismutase transgene (SOD1**^**G93A**^**) and wild-type (WT) lumbar spinal cord ventral horn.** The majority of tomato lectin (TL)-positive microglia (red) in the ventral horn did not express inducible nitric oxide synthase (iNOS) (arrows, A,B) at 6 weeks of age in either WT **(A)** or SOD1^G93A ^**(B)** mice. A subset of microglia were iNOS-positive (green) (arrows, C,D). The numbers of iNOS-positive and iNOS-negative microglia stayed relatively constant over time in WT mice **(E)**. The number of iNOS-positive microglia increased with disease progression in SOD1^G93A^ mice; the number of iNOS-negative microglia increased at 22 and 25 weeks of age in SOD1^G93A^ mice **(F)**. The combined data from Figure 2F and 3F show that the number of both arginase1 (Arg1)-positive and iNOS-positive microglia increased with disease progression **(G)**; the percentage of microglia expressing Arg1 and expressing iNOS also increased with time **(H)**. Scale bar 50 μm in A-D. *P < 0.05, **P < 0.01, ***P < 0.001. NY, Nuclear yellow; EP, endpoint.

Within the lumbar ventral horn, both microglial cells lacking iNOS (Figure 3A,B, arrows) and expressing iNOS (Figure 3C,D, arrows) could be observed. In WT mice there were more iNOS-negative than iNOS-positive microglia at all time points (Welch *t*-test *P* = 0.126 at 6 weeks; *t*-test *P* < 0.05 at 10 to 25 weeks; Figure 3E) with iNOS-positive microglia comprising less than 20% of total microglia. In SOD1^G93A^ mice the number of iNOS-expressing microglia showed an increasing trend beginning at 14 weeks, although the number of iNOS-positive microglia remained less than the number of iNOS-negative microglia up to 22 weeks of age (*t*-test *P* < 0.05 at 6 to 22 weeks) due to a concomitant increase in the number of iNOS-negative microglia (Figure 3F). Only at 25 weeks of age (disease endpoint) was there no difference between iNOS-positive (100 ± 54 microglia/mm^2^) and iNOS-negative microglia (281 ± 15 microglia/mm^2^, *t* test *P* = 0.069). By disease endpoint, the number of iNOS-expressing microglia had increased approximately seven times from the 10-week old SOD1^G93A^ lumbar spinal cord. A direct comparison between the number of Arg1-positive and iNOS-positive microglia in SOD1^G93A^ lumbar ventral horn (Figure 3G; data compiled from Figure 2F and Figure 3F) indicated that the number of Arg1-positive microglia was consistently greater than the number of iNOS-positive microglia at 18 weeks of age (130 ± 15 Arg1-positive microglia/mm^2^ versus 72 ± 13 iNOS-positive microglia/mm^2^, *t* test *P* = 0.040), and this difference was maintained till disease endpoint (Figure 3G). When the cell counts were expressed in terms of percentage of microglia expressing Arg1 or iNOS (Figure 3H), a similar trend was obtained. The percentage of SOD1^G93A^ lumbar microglia expressing Arg1 increased over time, from 14% at 10 weeks of age to 65% at 22 weeks of age. Over the same period, the percentage of SOD1^G93A^ lumbar microglia expressing iNOS increased from 8% to a peak of 33% (Figure 3H).

### SOD1^G93A^ lumbar microglia show increasing arginase1 and inducible nitric oxide synthase immunoreactivity with disease progression

**Figure 4:**
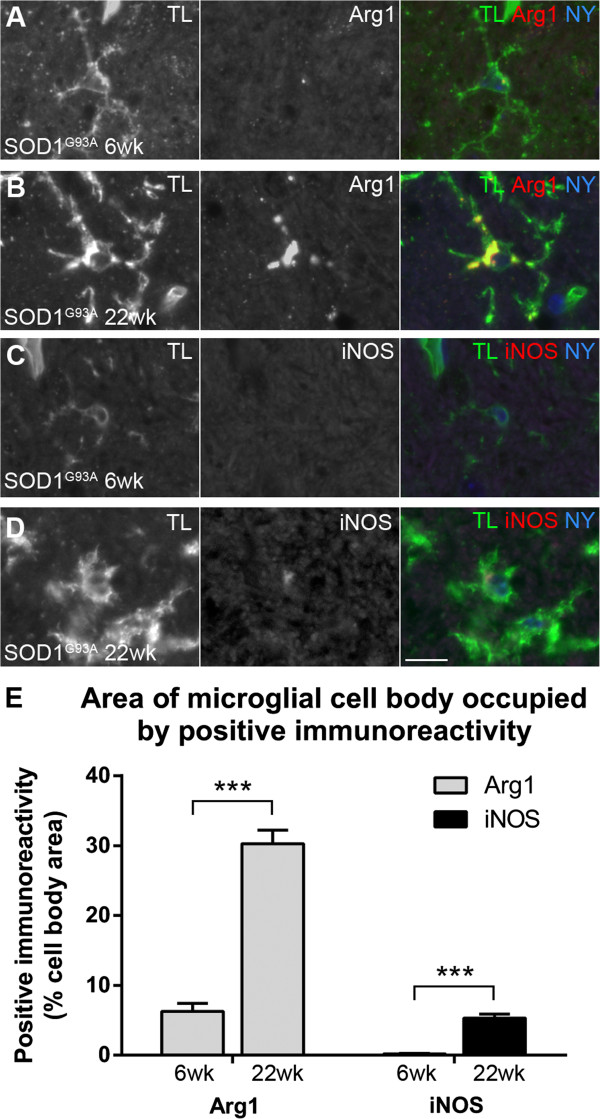
**Percentage area occupied by arginase1 (Arg1) and inducible nitric oxide synthase (iNOS) immunoreactivity in amyotrophic lateral sclerosis-linked mutant human superoxide dismutase transgene (SOD1**^**G93A**^**) lumbar spinal cord microglia.** The percentage area of the microglial cell body occupied by Arg1 **(A,B)** or iNOS **(C,D)**  positive immunoreactivity was examined at 6 weeks **(A,C)** and 22 weeks **(B,D)** of age in a subset of microglia displaying the strongest immunoreactivity. The percentage of cell body area occupied by Arg1 was higher at 22 weeks of age than at 6 weeks of age; the percentage of cell body area occupied by iNOS was also higher at 22 weeks of age than at 6 weeks of age **(E)**. Scale bar 10 μm in A-D. ***P < 0.01. TL, tomato lectin; NY, Nuclear yellow.

Ventral horn microglia from randomly selected lumbar spinal cord sections from SOD1^G93A^ mice showed an increase in the percentage area occupied by Arg1 and iNOS immunoreactivity between 6 weeks and 22 weeks of age (Figure 4). At 6 weeks of age, approximately 6% of the microglial cell body area was occupied by Arg1 immunoreactivity, whereas less than 1% of the cell body area contained iNOS immunoreactivity (Figure 4A,C,E). At 22 weeks of age, Arg1 immunoreactivity occupied approximately 30% of the cell body area, and iNOS immunoreactivity occupied around 5% of the cell body area (Figure 4B,D,E).

### Arginase1 and inducible nitric oxide synthase-expressing microglia in the cervical spinal cord of wild-type and SOD1^G93A^ mice

**Figure 5:**
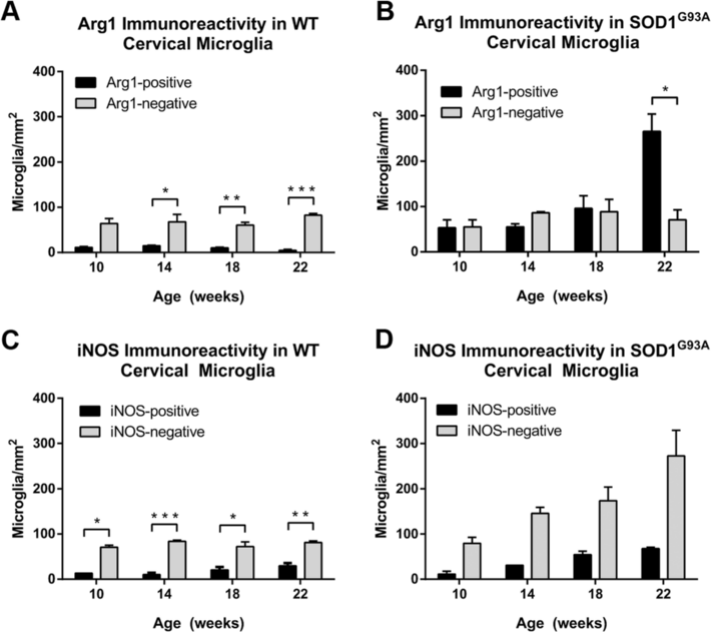
**Expression of arginase1 (Arg1) and inducible nitric oxide synthase (iNOS) in microglia of the ventral horn of wild-type (WT) and amyotrophic lateral sclerosis-linked mutant human superoxide dismutase transgene (SOD1**^**G93A**^**) cervical spinal cord.** The number of Arg1-negative and Arg1-positive cervical microglia remained relatively constant over time in WT mice **(A)**. In the cervical spinal cord of SOD1^G93A^ mice, the number of Arg1-positive and Arg1-negative microglia remained equivalent until 22 weeks of age, when there was an increase in the number of Arg1-positive microglia **(B)**. There were greater numbers of iNOS-negative microglia than iNOS-positive microglia throughout the time course in WT mice **(C)**. The numbers of iNOS-positive microglia and iNOS-negative microglia both increased with disease progression in the SOD1^G93A^ cervical spinal cord **(D)**. *P < 0.05, **P < 0.01, ***P < 0.001.

The current study focuses primarily on the histological changes in the lumbar spinal cord of SOD1^G93A^ mice, because motor weakness is first detectable in the hindlimbs in this mouse model. However, given the later involvement of the forelimbs [4,25] which are innervated by cervical motor neurons, we have made a brief comparison of microglial expression of putative inflammatory markers in the cervical spinal cord of WT and SOD1^G93A^ mice to determine whether temporal differences in Arg1 or iNOS expression between cervical and lumbar microglia may explain the differential involvement of the forelimbs and hindlimbs. In the WT mice, similar to the lumbar spinal cord, the numbers of Arg1-positive and Arg1-negative cervical microglia remained relatively constant over time (Figure 5A), with more Arg1-negative than Arg1-positive microglia at 14 weeks and after (*t*-test *P* < 0.05). In the SOD1^G93A^ cervical ventral horn, the number of Arg1-positive microglia was comparable to the number of Arg1-negative microglia up to 18 weeks, after which an increase in the number of Arg-positive microglia was recorded (265 ± 39 Arg1-positive microglia/mm^2^ versus 71 ± 22 Arg1-negative microglia/mm^2^, *t*-test *P* = 0.012; Figure 5B). The percentage of Arg1-positive microglia in the SOD1^G93A^ cervical ventral horn increased from 45% at 10 weeks of age to 79% at 22 weeks of age. Therefore, similar to the SOD1^G93A^ lumbar spinal cord, the cervical region also showed a trend of increasing numbers of Arg1-expressing microglia at 22 weeks of age.

With regard to iNOS labeling, the cervical spinal cord of WT mice showed a constant population of microglia, with more iNOS-negative than iNOS-positive microglia (Welch *t*-test *P* = 0.049 at 10 weeks; *t*-test *P* < 0.05 at 14 to 22 weeks; Figure 5C). In the cervical spinal cord of SOD1^G93A^ mice, the number of iNOS-positive microglia increased over time from 10 weeks of age (11 ± 7 iNOS-positive microglia/mm^2^) to 22 weeks of age (68 ± 3 iNOS-positive microglia/mm^2^) (Figure 5D). The number of iNOS-negative microglia also increased over the same time period (80 ± 13 iNOS-negative microglia/mm^2^ at 10 weeks versus 272 ± 57 iNOS-negative microglia/mm^2^ at 22 weeks of age) (Figure 5D). However, due to the use of the Welch *t*-test correction for unequal variances, there were no significant differences detected between iNOS-positive and iNOS-negative microglial numbers in the SOD1^G93A^ cervical spinal cord (Welch *t*-test *P* = 0.051 to 0.076 at 10 to 22 weeks). The percentage of iNOS-positive microglia in the SOD1^G93A^ cervical spinal cord increased from 11% at 10 weeks of age to 24% at 18 weeks of age, and 20% at 22 weeks of age.

### SOD1^G93A^ mice show a decreasing number of arginase1-negative motor neurons in the lumbar ventral horn as disease progresses

**Figure 6:**
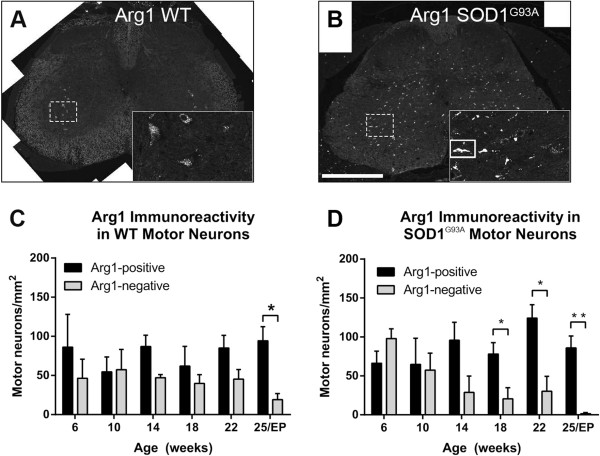
**Arginase1 (Arg1) expression in motor neurons of wild-type (WT) and amyotrophic lateral sclerosis-linked mutant human superoxide dismutase transgene (SOD1**^**G93A**^**) ventral horn.** Arg1 immunostaining was observed in a punctate pattern in motor neuron cell bodies (A, inset). At 22 weeks of age, Arg1 labeling was far more prominent in WT motor neurons **(A)** than in SOD1^G93A^ motor neurons **(B)**. The majority of Arg1-positive cells in the 22-week SOD1^G93A^ spinal cord were microglial cells (B; white box in inset). At 25 weeks of age, WT mice showed fewer Arg1-negative than Arg1-positive motor neurons **(C)**. In SOD1^G93A^ mice, there was an earlier decline in the number of Arg1-negative motor neurons, from approximately 18 weeks of age **(D)**, with significantly fewer Arg1-negative than Arg1-positive motor neurons at 18, 22 and 25 weeks of age (disease endpoint, EP). Scale bar 300 μm in A and B, 130 μm in A (inset), 125 μm in B (inset). *P < 0.05, **P < 0.01, ***P < 0.001.

In both SOD1^G93A^ and WT mice a number of motor neurons in the ventral horn of the lumbar spinal cord expressed Arg1 (Figure 6). The number of Arg1-positive motor neurons was not different between WT and SOD1^G93A^ mice at any time point measured (Figure 6C,D). In WT mice the number of Arg1-negative motor neurons stayed relatively constant from 6 to 22 weeks, but was decreased at 25 weeks (19 ± 8 Arg1-negative motor neurons/mm^2^), such that it was less than the number of Arg1-expressing motor neurons (94 ± 18 Arg1-positive motor neurons/mm^2^, *t*-test *P* = 0.018; Figure 6C). In comparison, the number of Arg1-negative motor neurons declined progressively in SOD1^G93A^ mice from 6 weeks of age (98 ± 13 Arg1-negative motor neurons/mm^2^) to 25 weeks of age (1 ± 1 Arg1-negative motor neurons/mm^2^) (Figure 6D). A difference between the number of Arg1-negative and Arg1-positive motor neurons in the SOD1^G93A^ lumbar spinal cord was first detected at 18 weeks of age (21 ± 14 Arg1-negative motor neurons/mm^2^ versus 78 ± 15 Arg1-positive motor neurons/mm^2^, *t* test *P* = 0.048), and the number of Arg1-negative motor neurons declined further until disease endpoint (Figure 6D).

### Ubiquitin pathology is present in SOD1^G93A^ lumbar spinal cord from 6 weeks of age

**Figure 7:**
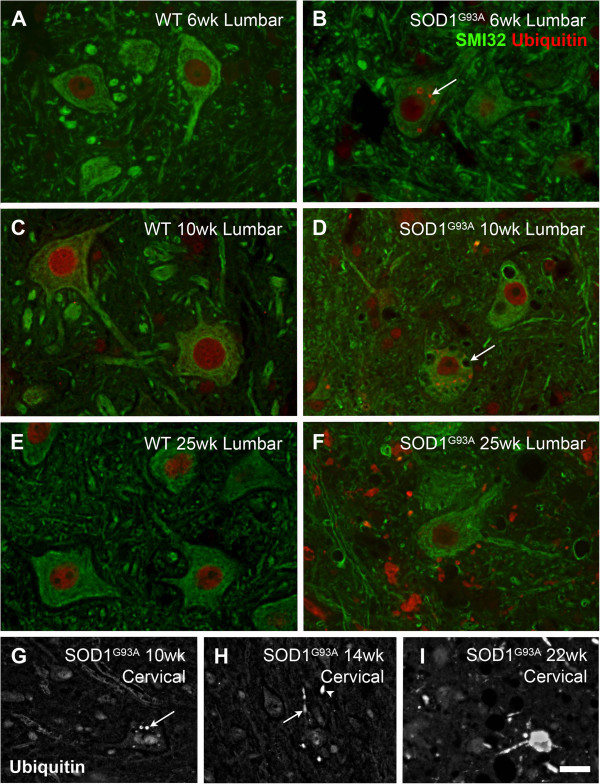
**Ubiquitin immunolabeling in amyotrophic lateral sclerosis-linked mutant human superoxide dismutase transgene (SOD1**^**G93A**^**) and wild-type (WT) mice.** Ubiquitin (red, A-F) was localized to the nucleus in WT **(A,C,E)** and SOD1^G93A^**(B,D,F)** motor neurons (SMI32, green, A-F). In SOD1^G93A^ mice lumbar spinal cord, intracellular ubiquitin-positive inclusions were present from 6 weeks of age (arrow, B) onwards. SOD1^G93A^ mice also showed extra-neuronal ubiquitin-positive inclusions from 10 weeks of age (D), with numerous ubiquitin aggregates by 25 weeks of age (F). Additionally, vacuolated lumbar motor neurons were observed from 10 weeks of age (arrow, D). Intracellular ubiquitinated aggregates were observed at 10 weeks of age in cervical motor neurons (**G**, arrow); at 14 weeks of age, intracellular (**H**, arrow) and extracellular (H, arrowhead) ubiquitin-positive aggregates were present in the SOD1^G93A^ cervical ventral horn, and increased in number with time (**I**, 22 weeks of age). Scale bar 12 μm in A-F, 20 μm in G-I.

Diffuse ubiquitin immunolabeling was present in the nucleus of both WT (Figure 7A,C,E) and SOD1^G93A^ (Figure 7B,D,F) lumbar motor neurons at 6, 10, 14 and 25 weeks of age (Figure 7). Ubiquitin-positive inclusions were present in the cytoplasm of SOD1^G93A^ motor neurons from as early as 6 weeks of age (Figure 7B, arrow). The number of ubiquitinated inclusions in the spinal cord increased with disease progression, with some non-neuronal ubiquitin immunoreactivity evident from 10 weeks of age (Figure 7D). By disease endpoint, numerous large ubiquitin-positive inclusions were located outside SMI32-positive motor neuron cell bodies in the gray matter (Figure 7F), and were also present in the white matter. These inclusions may be present in neuritic processes, glial cells or within the remnants of degenerating motor neurons. Additionally, immunolabeling for dephosphorylated neurofilaments (SMI32) revealed the presence of vacuolated lumbar motor neurons from 10 weeks of age in SOD1^G93A^ mice (Figure 7D, arrow). No such ubiquitin pathology or vacuolation was observed in WT motor neurons (Figure 7A,C,E).

In the SOD1^G93A^ cervical spinal cord, immunostained for ubiquitin at 10, 14 and 22 weeks of age (Figure 7G-I), cytoplasmic ubiquitin-positive inclusions were present in motor neurons from 10 weeks of age (Figure 7G, arrow), with non-neuronal ubiquitin aggregates distributed sparsely if present. At 14 weeks of age, ubiquitinated aggregates were observed in both soma of cervical motor neurons and within the neuronal processes of the neuropil (Figure 7H, arrow) or as an extracellular aggregate (Figure 7H, arrowhead). Ubiquitin pathology increased with disease progression in the cervical spinal cord as in the lumbar spinal cord; by 22 weeks of age, multiple non-neuronal ubiquitinated aggregates were present in the cervical spinal cord (Figure 7I). The presence of ubiquitin pathology appears to be slightly delayed in the cervical spinal cord compared to the lumbar spinal cord of SOD1^G93A^ mice.

### As disease progresses, SOD1^G93A^ mice differ from wild-type mice in body weight, outcome of wire-hang test, stride length and uniformity measurement

#### Survival

Average survival time of the SOD1^G93A^ mice was 164 ± 2 days and median survival (50%) was 166 ± 2 days. All SOD1^G93A^ mice reached disease endpoint as determined by weight loss (a loss of 20% from their maximum body weight) before losing their righting reflex. All WT mice remained healthy at the endpoint ages of their SOD1^G93A^ littermates.

#### Body weight

**Figure 8:**
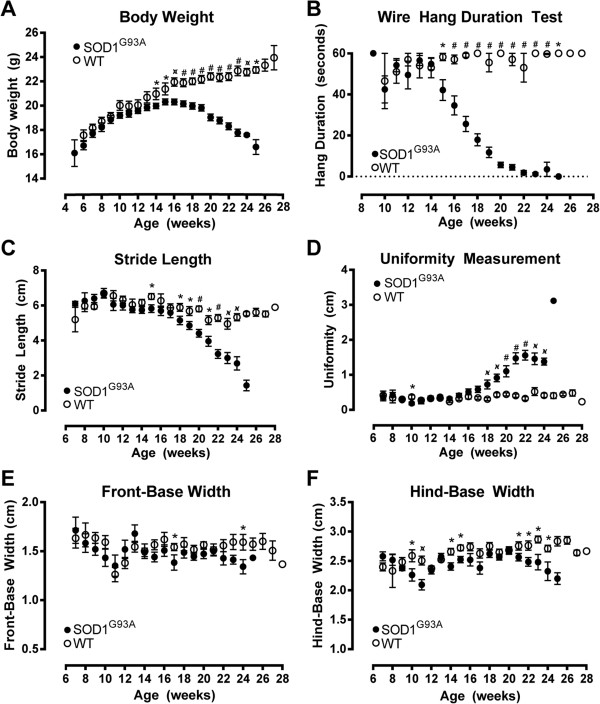
**Functional measures of disease progression in wild-type (WT) and amyotrophic lateral sclerosis-linked mutant human superoxide dismutase transgene (SOD1**^**G93A**^**) mice.** Body weight increased continuously in WT mice but followed a curved trajectory in SOD1^G93A^ mice, becoming significantly less than that of WT mice at 14 weeks of age and reaching peak body weight at approximately 16 weeks of age **(A)**. WT mice were able to maintain wire hang duration throughout the study, while SOD1^G93A^ mice became unable to maintain a 60-second hang duration from 15 weeks of age onwards **(B)**. SOD1^G93A^ mice displayed a lower stride length **(C)** and a higher uniformity measure **(D)** than WT mice at 18 weeks and thereafter. Front-base width **(E)** and hind-base width **(F)** showed no consistent changes between WT and SOD1^G93A^ mice over time. *P < 0.05, *P* < 0.01, ^**#**^*P* < 0.001.

Body weight rose continuously for WT mice throughout the duration of the study. In contrast, the SOD1^G93A^ mice displayed a curved body weight trajectory, achieving maximal weight (20.3 ± 0.2 g) at 16 weeks of age (Figure 8A). The average body weight of SOD1^G93A^ mice was lower than that of WT mice from the age of 14 weeks (20.0 ± 0.2 g and 21.0 ± 0.5 g respectively, MWU *P* = 0.035) onwards, with the body weight difference increasing with disease progression.

#### Wire-hang test

WT mice were able to maintain wire hang duration time on inverted wire bars for 60 seconds (the set maximum time) throughout the course of the study, while SOD1^G93A^ mice were only able to maintain similar hang duration until 14 weeks of age (Figure 8B). At 15 weeks, SOD1^G93A^ mice recorded lower wire-hang times than WT mice (42 ± 5 seconds and 58 ± 2 seconds, respectively, MWU *P* = 0.012), and by 20 weeks SOD1^G93A^ mice could only achieve a wire-hang time of less than 10 seconds (Figure 8B). As an interesting aside, we noted that the first 3 to 4 weeks of testing were highly variable for both SOD1^G93A^ and WT mice. We attribute these variable measurements to a learning effect for these mice, whose home cages had a plastic lid - the only wire structure in the home cage was the food hopper, hence these mice had no prior experience clinging onto a set of wire bars while inverted.

#### Stride length

The average stride length of WT mice stayed relatively constant over time, while the stride length of SOD1^G93A^ mice declined from approximately 17 weeks of age (Figure 8C). The stride length of SOD1^G93A^ mice was less than that of WT littermates at 18 weeks (5.2 ± 0.2 cm and 5.9 ± 0.2 cm, respectively, MWU *P* = 0.021), with the difference between the two groups increasing progressively over time thereafter.

#### Uniformity

Uniformity measurement, the distance between placement of the hind paw compared with the front paw from the previous step, differed between WT and SOD1^G93A^ mice over time (Figure 8D). WT mice maintained a uniformity distance of less than 0.5 cm throughout the study; that is, hind paw placement was within 0.5 cm of the front paw placement on the previous step. However, this began to differ between genotypes at 18 weeks of age, with SOD1^G93A^ mice showing a larger uniformity measurement (SOD1^G93A^ 0.7 ± 0.1 cm versus WT 0.3 ± 0.1 cm, MWU *P* = 0.002). The difference between the two groups was maintained from 18 weeks to 24 weeks of age (Figure 8D); the single uniformity value obtained from the one surviving SOD1^G93A^ mouse at the 25-week time point was insufficient to perform statistical analysis. The increase in uniformity in SOD1^G93A^ mice likely reflects the diminished strength of the hindlimb with disease progression, and resultant inability to move the hindlimb forward efficiently during walking.

#### Front-base width and hind-base width

Front-base width did not differ between WT and SOD1^G93A^ mice, apart from the isolated time points at 17 and 24 weeks (Figure 8E). Similarly, for most time points, WT and SOD1^G93A^ mice showed similar values of hind-base width (Figure 8F). SOD1^G93A^ mice showed a slight decline in hind-base width over time from 21 weeks onwards; however this was still within the range of measurements obtained at earlier time points (Figure 8F).

## Discussion

ALS is a neurodegenerative disease affecting the motor system, with disease progression thought to be influenced by inflammatory responses in the spinal cord. A main finding of this study was that the total number of microglia in the ventral horn of the SOD1^G93A^ lumbar spinal cord, from 14 weeks of age, was mainly associated with an increase in Arg1-positive microglia (Figures 1 and 2). Increasing numbers of iNOS-expressing microglia were also demonstrated in the ventral horn (Figure 3), while the number of motor neurons lacking Arg1 declined sharply from 18 weeks of age in the SOD1^G93A^ spinal cord (Figure 6). A similar trend of increasing Arg- and iNOS-expressing microglia over time appeared to be present in the SOD1^G93A^ cervical spinal cord (Figure 5). Additionally, we found that ubiquitin pathology was detected in the lumbar spinal cords of 6-week old SOD1^G93A^ mice (Figure 7), before any observable functional decline (Figure 8).

### Microgliosis in the SOD1^G93A^ spinal cord

Our results demonstrated a substantial increase in the number of microglial cells in the SOD1^G93A^ mouse lumbar spinal cord, starting from symptom onset at 14 weeks, and increasing with disease progression through to endpoint (Figure 1). This result is consistent with previous reports in the literature, describing an increase in the number of activated microglia in spinal cord gray matter at disease onset, and increasing throughout disease progression [11,12,18,26-28]. A few previous reports have also demonstrated an increase in the number of microglia during the pre-symptomatic stage in SOD1^G93A^ mice [10], SOD1^G93A^ rats [29] and SOD1^H46R^ rats [30]. These studies may show earlier increases in microglia due to the use of CD11b [10,29], perhaps a more sensitive marker of microglia than the TL used in the present study. Additionally, it is possible that the SOD1^H46R^ rat model may exhibit a slightly different disease course with altered timing of microglial activation compared with the SOD1^G93A^ rodent models, as differences in the survival time and disease severity between SOD1^G93A^ and SOD1^H46R^ mouse models have been previously reported [31]. Alternatively, the pre-symptomatic microglial activation seen in previous studies may be a response to factors released by dysfunctional motor neurons before the onset of overt functional deficits. Given that in this study we saw the presence of intracellular ubiquitinated aggregates as early as 6 weeks of age in the lumbar spinal cord, while microgliosis was observed from 14 weeks of age, the data indicate that the initial microgliosis might be a reactive response to the presence of motor neuron dysfunction and degeneration in the lumbar spinal cord. Multiple lines of evidence indicate that microglial activation plays a key role in the progression of disease after onset [12,32,33], and indeed we observed that obvious changes in functional ability of the SOD1^G93A^ mice, such as the alterations to stride pattern parameters, occurred at 18 weeks of age - 4 weeks after the initial increase in the number of microglia in the lumbar spinal cord. Thus, the increase in microglial numbers preceded the transition into a phase of disease progression in which functional ability declined rapidly.

### Phenotype of microglia in the SOD1^G93A^ spinal cord

We found a seven-fold increase in the number of iNOS-positive microglia between the pre-symptomatic stage and disease endpoint, which translated to an increase in the percentage of microglia expressing iNOS from less than 10% pre-symptomatically to over 30% at 22 weeks of age (Figure 3). Interestingly, the number of lumbar spinal cord microglia expressing Arg1 also increased at disease onset and continued to increase throughout disease progression, with an 18-fold increase at endpoint in comparison to pre-symptomatic levels; similarly, the percentage of microglia expressing Arg1 increased from below 20% in pre-symptomatic lumbar spinal cord to over 60% at endpoint (Figures 2 and 3). The increasing numbers of iNOS-expressing and Arg1-expressing microglia in the SOD1^G93A^ lumbar spinal cord over time were surprising, given that both Arg1 and iNOS have competitive roles in L-arginine metabolism. The increase in the number of Arg1-expressing lumbar microglia as disease progresses may be an attempt to limit tissue damage and neuronal degeneration caused by disease processes [20,34], but these results did not appear to be in line with the widely-perceived switch from an M2 to an M1 phenotype in SOD1^G93A^ mice [12,13].

To examine whether the expression levels of Arg1 or iNOS within individual microglia were altered during disease progression, a semi-quantitative analysis of protein expression level was carried out using sections immunostained for Arg1 and for iNOS (Figure 4). This analysis showed that, in Arg1-positive microglia, the amount of Arg1 immunoreactivity increased with disease progression; the same was true for iNOS-positive microglia showing an increase in iNOS immunoreactivity (Figure 4). The percentage area occupied by Arg1 immunoreactivity increased approximately five-fold between 6 and 22 weeks of age, yet the percentage area occupied by iNOS immunoreactivity increased approximately 25-fold between 6 and 22 weeks of age due to the small amount expressed at 6 weeks of age (Figure 4). Conversely, while the absolute levels of Arg1 and iNOS protein cannot be reliably compared by immunohistochemistry intensity due to different antibody-antigen binding strengths, the percentage area occupied by Arg1 does appear to be larger than that occupied by iNOS. Thus, our results show evidence of both neurotoxic and neuroprotective processes in SOD1^G93A^ spinal cord microglia, as implicated by increasing immunoreactivity for iNOS and Arg1, respectively, over time.

Previous studies have reported the upregulation of M2 microglial markers such as Ym1 and MCP-1 in SOD1^G93A^ mouse spinal cord during the early, slow-progressing phase of disease [12,32]. Subsequently during the rapidly-progressing disease phase, M2 markers were downregulated and the M1 markers tumor necrosis factor α, NOX2 and IL-6 became more prominent [12,13,18,32,35,36]. While the increase in iNOS immunoreactivity in SOD1^G93A^ microglia with disease progression in this study would agree with the upregulation of M1 markers, we saw no concomitant decrease in expression of the M2 marker Arg1. The differences between our study and previous studies may be due to the use of different techniques to assess microglial markers; the current study used immunohistochemistry to reveal cell-specific localization data, compared with mRNA or protein data from homogenized spinal cord. Although microglia are very likely to be the primary cell type responsible for cytokine production within the spinal cord, other cell types including astrocytes and motor neurons may also produce cytokines or inflammatory markers which may skew the results of mRNA studies. Indeed, in the current study, we observed that spinal cord motor neurons expressed the putative M2 marker Arg1 (Figure 5). Furthermore, it has been shown that certain M2 markers such as Ym1 increased in endstage SOD1^G93A^ mice [37]. Additionally, Chiu and colleagues demonstrated that SOD1^G93A^ microglia can show M2 characteristics, such as increased production of insulin-like growth factor 1 and decreased IL-6 expression, regardless of disease progression stage [26].

The use of mRNA from homogenized spinal cord alone may limit interpretation of which proteins are specifically present in microglial cells. On the other hand, the use of protein markers alone in this study resulted in a different issue: we were unable to perform reliable double-labeling for Arg1 and iNOS together due to persistent antibody cross-reactivity. Thus, some microglia expressing Arg1 may also have expressed iNOS. Additionally, microglial phenotype is regulated by a number of pro- and anti-inflammatory cytokines, some of which (interferon γ, IL-4 and IL-10 [34]) have a direct effect on Arg1 and iNOS activity [38], so it is conceivable that upregulation of Arg1 and iNOS in subsets of microglia could occur as a response to the cytokines in the external milieu of the microglia. Alternatively, the regulation of iNOS and Arg1 levels may occur independently of the classical M1/M2 phenotypes driven by changes in cytokine production.

### Comparison of arginase1 and inducible nitric oxide synthase protein expression in the SOD1^G93A^ lumbar and cervical spinal cord

We compared the cervical and lumbar regions of the spinal cord and investigated whether changes in Arg1 or iNOS expression could explain the delayed involvement of the forelimbs in the SOD1^G93A^ mouse model. In the SOD1^G93A^ cervical ventral horn, the number of Arg1-positive microglia was increased at 22 weeks of age, while the number of iNOS-positive microglia also increased slightly over time (Figure 5). Although these trends were similar to those seen in the lumbar spinal cord (Figures 2 and 3), a few small differences may indicate a slightly different microglial environment in the cervical spinal cord. First, at 10 weeks of age there were equivalent numbers of Arg1-positive and Arg1-negative microglia in the cervical spinal cord compared with a predominance of Arg1-negative microglia (86%) in the lumbar spinal cord, possibly indicating the existence of a less inflammatory microenvironment in the cervical spinal cord. Second, the percentage of microglia expressing Arg1 reached 79% at 22 weeks of age in the cervical spinal cord, compared with 64% in the lumbar spinal cord. Furthermore at 22 weeks, the percentage of microglia expressing iNOS was 24% in the cervical spinal cord, compared with 32% in the lumbar spinal cord. Taken together, these data may indicate that a more neuroprotective microglial phenotype is present in the cervical than the lumbar spinal cord during the time course of the disease. It is difficult to elucidate from this study whether the apparently less inflammatory microglial environment in the cervical spinal cord is due to delayed neuronal degeneration, or *vice versa*. However, in line with a previous study comparing cervical and lumbar spinal cord regions, our results indicate that the cervical spinal cord showed increased early production of an M2 marker compared to lumbar spinal cord in the SOD1^G93A^ mouse model [12].

### Arginase1 protein expression in motor neurons

We examined the change in Arg1-negative and Arg1-positive motor neurons over time in the SOD1^G93A^ lumbar spinal cord. The number of Arg1-negative motor neurons decreased substantially after disease onset in SOD1^G93A^ mice, whereas the number of Arg1-positive motor neurons remained relatively constant (Figure 6). Motor neurons lacking Arg1 may be more vulnerable compared with motor neurons which express Arg1, possibly due to NO toxicity. Motor neurons in SOD1^G93A^ mice are reported to have decreasing Arg1 expression [19] and increasing iNOS expression [39] with disease progression and can be protected by L-arginine supplementation [19]. The decreasing level of Arg1 and increasing level of iNOS may disrupt the normal balance of arginine metabolism, with SOD1^G93A^ motor neurons skewed towards iNOS-mediated production of NO rather than Arg1-mediated production of ornithine [40-44]. Increased production of NO, mediated by Arg1 inhibition, is toxic to motor neurons *in vitro*[45-47]. In addition to raising NO levels, low levels of Arg1 could also decrease production of neuroprotective polyamines such as spermine and spermidine, normally produced from ornithine [19,48]. Thus, SOD1^G93A^ motor neurons with low levels of Arg1 may be more susceptible to toxicity due to their higher levels of NO and lower levels of neuroprotective polyamines.

### Functional decline and ubiquitin pathology

The current study confirmed the decline in function over time in SOD1^G93A^ mice, which has been well documented previously [4]. Our SOD1^G93A^ cohort showed divergence from their WT littermates in body weight and in wire hang duration ability from 14 and 15 weeks of age, respectively, with overt changes in stride parameters becoming apparent at 18 weeks of age (Figure 8). We therefore considered 14 weeks of age to be the point of disease onset, for comparison with spinal cord changes observed by immunohistochemistry. Our estimate of 14 weeks of age is conservative compared to previous studies in the SOD1^G93A^ mouse on a C57BL/6 background, showing initial onset of disease symptoms detectable at 11 weeks of age by observations of tremors [12,32]. However, the development of overt stride parameter differences at 18 weeks of age in the current study correlates well with the transition into a rapidly progressing phase of disease at approximately 18 weeks of age, as measured using the BASH scoring system [32].

Additionally, in this study, we have shown that ubiquitin pathology and vacuolated motor neurons were present in the lumbar spinal cord of SOD1^G93A^ mice at 6 weeks and 10 weeks of age, respectively (Figure 7). The presence of vacuolated motor neurons and ubiquitinated aggregates in pre-symptomatic SOD1^G93A^ mice has been reported previously [49,50]. Compared with our detection of disease onset at 14 weeks of age (Figure 8), the presence of ubiquitin and neurofilament pathology in SOD1^G93A^ lumbar motor neurons from 6 weeks of age onwards reinforces the idea that pathological changes in the motor neuron cell body occur well before onset of functional deficits, due to the compensatory peripheral plasticity [51]. The later appearance of pathological changes in the cervical spinal cord correlate with the reported later involvement of the forelimbs compared with the hindlimbs in the SOD1^G93A^ mice [25]. Unfortunately, in this study we were not able to directly correlate delayed cervical pathology with functional deficits, as our measures of stride length, uniformity and wire hang duration are all related to hindlimb function.

## Conclusions

In summary, we have shown that the numbers of microglia expressing the M2 marker Arg1, as well as the numbers of microglia expressing the M1 marker iNOS, increase in the spinal cord during disease progression in the SOD1^G93A^ mouse model of ALS. It is likely that evaluation of the cytokine protein profile will be key to understanding the overall neuroprotective or neurotoxic effects of microglial activation on the spinal cord milieu. We have also demonstrated a selective decrease in motor neurons lacking expression of Arg1, and strategies to boost Arg1 expression may also be an appropriate therapeutic target for treatment of human ALS. Modulation of microglial immune responses remains an important target for ameliorating disease symptoms and the rate of disease progression in ALS.

## Abbreviations

ALS: amyotrophic lateral sclerosis; Arg1: arginase1; Iba1: anti-ionized calcium binding adaptor molecule 1; IL: interleukin; iNOS: inducible nitric oxide synthase; MOM: Mouse on Mouse; MWU: Mann Whitney *U* test; NO: nitric oxide; PBS: phosphate buffered saline; PFA: paraformaldehyde; SOD1: superoxide dismutase 1; SOD1G93A: ALS-linked mutant human superoxide dismutase transgene; TL: tomato lectin; WT: wild-type.

## Competing interests

The authors declare that they have no competing interests.

## Authors’ contributions

KEL carried out the immunohistochemistry (iNOS, Arg1, TL), cell counts, functional assessment, statistical analyses and preparation of the manuscript. ALR carried out the immunohistochemistry (iNOS, Arg1, TL and Iba1). WB and AKW participated in the design and data analysis of the study. AK carried out the immunohistochemistry (ubiquitin) and contributed to the preparation of the manuscript. RSC conceived of the study, participated in the design and data analysis. MIC conceived of the study, contributed to the design, assisted in functional assessment and preparation of the manuscript. All authors read and approved the final manuscript.
